# Mental health, attachment and breastfeeding: implications for adopted children and their mothers

**DOI:** 10.1186/1746-4358-1-5

**Published:** 2006-03-09

**Authors:** Karleen D Gribble

**Affiliations:** 1 School of Nursing, Family and Community Health, Locked Bag 1797, Penrith South DC, NSW, 1797, Australia

## Abstract

Breastfeeding an adopted child has previously been discussed as something that is nice to do but without potential for significant benefit. This paper reviews the evidence in physiological and behavioural research, that breastfeeding can play a significant role in developing the attachment relationship between child and mother. As illustrated in the case studies presented, in instances of adoption and particularly where the child has experienced abuse or neglect, the impact of breastfeeding can be considerable. Breastfeeding may assist attachment development via the provision of regular intimate interaction between mother and child; the calming, relaxing and analgesic impact of breastfeeding on children; and the stress relieving and maternal sensitivity promoting influence of breastfeeding on mothers. The impact of breastfeeding as observed in cases of adoption has applicability to all breastfeeding situations, but may be especially relevant to other at risk dyads, such as those families with a history of intergenerational relationship trauma; this deserves further investigation.

## Background

The value of breastfeeding in supporting the normal growth and development of infants and young children is recognized worldwide [1]. There is also a growing awareness that it is possible for women to breastfeed their adopted children, and that health care professionals should support them if they express a desire to do so [2]. However, both professional and lay literature are often unclear as to why adoptive breastfeeding may be of benefit. The ability of adoptive mothers to successfully relactate/induce lactation is regularly questioned in literature [3-5], in spite of evidence that most adoptive mothers are physiologically capable of producing sufficient milk for their child [6]. In addition, discussion of the benefits of adoptive breastfeeding uniformly lacks detail on how breastfeeding may assist the child or mother [3-5,7]. Thus, health care professionals and prospective adoptive parents may be left with the impression that adoptive breastfeeding is something that is nice to do but is without potential for substantive benefit.

In an attempt to ameliorate this situation, this paper will provide evidence to support the proposition that breastfeeding can play a significant role in facilitating the development of the child-mother relationship in cases of adoption. This evidence will include discussion of the impact of the physical act of breastfeeding on children and mothers; the way in which the pre-adoption experience of children influences their ability to form relationships; how breastfeeding may be initiated in cases of adoption and the potential impact of breastfeeding on adopted children. Four case histories of adoptive breastfeeding will also be presented.

### Non-nutritional aspects of breastfeeding

The positive impact of breastmilk on the growth and development of babies is widely accepted [8]. However, breastfeeding is more than just the provision of nutrition; the act of suckling at the breast has an impact on both child and mother. Breastfeeding calms and provides analgesia to infants, as evidenced in reduced heart and metabolic rates and a reduced ability to perceive pain during suckling [9-11]. There are several reasons for this calming and analgesic effect. Firstly, suckling at the breast stimulates the infant's oropharyngeal tactile- and mechanoreceptors and focuses attention on the mouth, reducing outside influences [12,13]. Secondly, suckling and intestinal adsorption of fat from milk stimulates the release of the hormone cholecystokinin [14,15], which activates an afferent vagal mechanism that induces relaxation and pain relief [16]. Thirdly, the sweet flavour of milk stimulates the release of opioids in the midbrain of infants, which act on receptors that decrease the perception of pain [10,17-19]. Fourthly, breastfeeding involves maternal skin-to-skin contact, which stabilises blood glucose levels [20], body temperature and respiration rates [20-22], aids neurobehavioral self-regulation [23] and also reduces stress hormone release [24] and blood pressure [25]. Finally, breastfeeding involves intimate social interaction between mother and child, which may result in the release of the anti-stress hormone oxytocin [26,27]. These mechanisms of relaxation and analgesia work synergistically [28,29] and while research has thus far focused on newborns, the experience of mothers is that the calming, analgesic and relaxing effects of breastfeeding remain for as long as breastfeeding occurs [30].

Breastfeeding also has an impact on mothers. The physiology and behaviour of mothers are influenced by the release of the hormones oxytocin, prolactin and cholecystokinin during breastfeeding. Oxytocin is released from the hypothalamus in response to skin-to-skin contact and suckling at the breast [31,32]. Oxytocin release during breastfeeding ejects milk from the myoepithelial cells in the breast allowing the infant to extract milk [32]; it also acts on the mother's central nervous system, causing physiological as well as behavioural changes in her [31-33]. Oxytocin is known to be essential for expression of maternal behaviour in some mammals [34,35] and, although the research is as yet scant, there is some evidence that oxytocin is also involved in the development of maternal love in humans [36]. Oxytocin also has a potent anti-stress impact on mothers [32,33].

The hormone prolactin is released from the pituitary in response to nipple stimulation, such as that occurring during suckling [37]. Prolactin is involved in milk production [37] and is thought to act on the central nervous system to promote maternal behaviour [33]. Prolactin acts as an analgesic and reduces stress responsiveness in animal models [33,38] and may well operate in a similar way in humans [33].

Cholecystokinin is released in infants as they suckle but it is also released in the intestine of mothers during breastfeeding [14]. Cholecystokinin is thought to promote maternal behaviour [39] and, as with infants, it relaxes and provides analgesia to mothers [40]. Oxytocin, prolactin and cholecystokinin act synergistically in mothers [33,40].

There is evidence that the hormonal and other effects of breastfeeding influence the physiology and behaviour of mothers. Breastfeeding women have lower blood pressure and respond less to emotional and physical stress than non-breastfeeding women [32,41-44]; they also show a "relaxation response" in their brains during breastfeeding [45]. Mothers who are less stressed are able to be more responsive to their babies [46,47], and it is therefore not surprising that some studies have found that breastfeeding women are more socially interactive [48] and exhibit a greater responsiveness and caring to their babies than non-breastfeeding mothers [49,50].

The increased responsiveness of breastfeeding women to their infants is perhaps a result of the hormonal influences already described, but it may also be related to the physical closeness of breastfeeding. Breastfeeding requires frequent close physical contact between mother and child [51,52] and some research has found that breastfeeding women seek greater proximity to their babies [53,54]. Breastfeeding involves infant-mother skin-to-skin contact which both increases a mother's desire to be with her baby [55,56] and her sensitivity to her infant [57,58]. Research has found that the more that babies and mothers are kept together, the greater the impact on the mother in terms of exhibition of responsive caregiving [59] and security of attachment in the child [60]. As will be discussed, development of attachment security is of great value to children [61,62].

### The experience of children placed for adoption

Before the potential impact of breastfeeding on adopted children can be understood the experience of children placed for adoption must be explored. Adopted children have all experienced the loss of their birth mother. Many adopted children have also experienced institutionalisation or hospitalisation; abuse or neglect at the hands of caregivers; or placement with multiple different caregivers. These experiences can all affect the ability of children to trust and build healthy relationships with their adoptive mothers. This paper will discuss relationship development between mother and child because it is the mother who is most often the primary caregiver and breastfeeding is a maternal role.

#### The losses associated with adoption

Adoption universally involves loss. Babies recognize their mothers at birth and at delivery healthy babies placed on the abdomen of their mother will crawl up onto her chest and, locating the nipple via its familiar smell [63], will attach to her breast and suckle [64,65]. Newborn infants desire to remain with their mother and if removed from skin-to-skin contact with her will give a specific "separation distress cry/call" as an appeal for reunion [66]. Maternal separation is stressful for infants [25,66-69], and all adopted children have experienced the loss of their birth mother. Some believe that this loss can impede the development of later relationships even where the birth mother is quickly substituted with another caregiver [70]. Nonetheless, children whose birth mothers are immediately replaced by adoptive mothers are not at as great a risk of experiencing difficulties with relationships as children whose histories include protracted relational trauma.

Some children placed for adoption do not have a smooth transition from loving care by their birthmother to loving care by adoptive parents; it is these children who are most at risk of difficulties with building relationships and may benefit most from adoptive breastfeeding. While children adopted as newborns may certainly be assisted by breastfeeding, the following discussion will focus on the most vulnerable children, those adopted from institutions or foster care after a history of abuse or neglect.

#### Children adopted after institutionalisation or hospitalisation

The number of children adopted worldwide via intercountry adoption is increasing each year and 20 000 children were adopted to the United States alone in 2004 [71]. Based on what is known of the practices in the countries from which children are adopted it is likely that a large proportion of intercountry adopted children lived in institutional care prior to adoption [72]. Although institutions vary widely in the quality of care they provide, they are often lacking in heating, cooling, space, toys or nutrition, and provide a restricted and regimented environment [72-75]. Institutions usually have high child to caregiver ratios, which do not allow for individualized attention [74,76], and children are generally cared for by many different adults [72]. The physical and emotional deprivations of institutionalisation can result in a raft of problems including physical and developmental delays, and language and sensory integration issues [74,77-79]. However, it can be argued that the most serious deprivation of institutionalisation is the lack of a consistent and sensitive caregiver whom a child can trust and form a healthy attachment to. Absence of a responsive primary caregiver is a type of relational trauma that alters and retards brain development [80,81], reduces capacity to deal with stress [81] and makes it more difficult for children to form close and trusting relationships [82].

A proportion of children placed for adoption have spent significant periods of time in hospital because of prematurity, illness or disability. Short-term hospitalisation with parental contact may be relatively benign [83]; however, long-term hospitalisation can have a sustained negative impact on children [84-87]. Children adopted after long-term hospitalisation have often spent critical months of infancy or toddlerhood without the care of a parent or other primary caregiver [87] and while hospital staff may meet the physical and medical needs of children they may not be able to provide for their emotional needs [87,88]. Under these circumstances, long-term hospitalisation is a form of institutional care in which children experience institutional neglect.

#### Children adopted from foster care

In all developed countries there are a large number of children who cannot be cared for by their parents and are therefore placed in foster care [89-92]. Children adopted from foster care often have a history of abuse or neglect [93-96] and many have also experienced multiple foster care placements [97]. Children adopted from foster care are vulnerable to having difficulty developing healthy relationships because of their experience of relationship failure [98-100].

### How relationship trauma affects the development of later relationships

#### The importance of attachment relationships

A history of abuse or neglect negatively affects the ability of children to trust and form relationships with their adoptive parents. Such children are said to be at risk of having attachment difficulties. Attachment is a close affectional bond, a reciprocal relationship that endures over time [101]. Ideally, a child will develop a secure attachment with a primary caregiver through mutual interaction in which the caregiver repeatedly gratifies his or her needs in an appropriate manner, resulting in reduction of anxiety/discomfort and in feelings of relaxation and relief [102]. This repeated gratification in response to need is called the attachment cycle [103]. The primary attachment relationship forms a base from which children explore themselves, others and the world; thus, the quality of attachment can impact the emotional, social and even physical development of children [104]. Children who have experienced consistent, responsive caregiving develop a positive internal working model of relationships and a high level of personal value, both of which are building blocks for success in future relationships [103,105,106]. Such children view the world as a safe place and are likely to cope well with confronting situations, confident that assistance, nurturance and protection will be available if and when needed [105].

However, children who are institutionalised, hospitalised, neglected or abused by caregivers or have experienced multiple placement often have a greatly reduced or disrupted experience of the attachment cycle. Children who are institutionalised do not have the attachment cycle completed frequently or consistently because of the ability of too few caregivers to meet the individual needs of many children [82]. This does not constitute a single stress but is repeated, prolonged and chronic traumatic stress [107]. Institutionalised children are also hampered in development of attachment security because they have multiple caregivers [82,108] and children can only build attachments with a small number of caregiving adults [109]. Similarly, children who are hospitalised long-term may not have individualised care or a primary caregiver [82]. In addition, a proportion of hospitalised children experience chronic unrelieved pain and such children cannot have the attachment cycle completed because the source of their distress cannot be removed [110]. This places them at even greater risk of being unable to trust a caregiver [111].

Children who are neglected may not have the attachment cycle completed or may have it completed inconsistently preventing them from trusting their caregiver and developing a secure attachment [112]. Those children who are abused by their primary caregiver have the attachment cycle disrupted by abuse and, in consequence, closeness with their caregiver results in fear rather than safety or comfort [82,112]. Children born drug addicted have experienced prenatal abuse [111], which can result in neurological impairment and cause irritability, pain and hypersensitivity to touch [113] making it difficult for the attachment cycle to be completed. The multiple placement that is experienced by many children in the foster care system hampers attachment security development because children lack a consistent caregiver. The more placements and changes in caregivers that children experience the more difficult it is for them to believe that they are not going to be abandoned again [114].

A child's preplacement experience will affect the development of the relationship between child and mother after adoption, because of the effect of past relationships on the child's internal working model of relationships. Children whose caregiving has been neglectful (including institutional neglect) or abusive (including multiple placement) have experienced relational trauma and are likely to have a negative working model of relationships, have low self worth and consider the world an unpredictable and hostile place [103,105,106]. Children with a history of relational trauma may experience lifelong difficulties with feeling empathy, trusting others and developing intimate relationships [81,115] as their internal model of relationships tells them, "Do not let us care too much for anyone. At all costs let us avoid any risk of allowing our hearts to be broken again" [[116], p124].

#### How a history of relational trauma manifests in the behaviour of children

For parents who have adopted a child with a history of abuse or neglect, the need of their child to protect themselves from further hurt can prove a challenge to the development of the parent-child relationship and a barrier to healing. This need for self-protection is manifest in various behavioural strategies that are adaptations to the child's life experience [78,82,100] including: exhibition of indiscriminate affection, avoidance of physical touch or eye contact, seeking to control relationships, premature independence and resistance to nurturing.

Children who have been institutionalised or who have experienced multiple placement are commonly indiscriminately affectionate and seek to engage and show physical affection to strangers [74,95,117]. In post-institutionalised children this behaviour may arise out of the experience of needing to be proactive in seeking scarce adult attention [74,118]; in children with a history of caregiver loss, it may be an indication of their expectation of future loss and a mechanism of seeking an alternative caregiver. Indiscriminately affectionate behaviour is problematic because it is often at the expense of interaction with parents [103]. It is not uncommon for children to refuse to make eye contact with parents, avoid physical contact or be stiff while being held [103]. Also, while children have experienced limited human touch or physical abuse and are in particular need of nurturing touch, many are touch adverse [98,103].

Children may also seek to control the relationship with their parents, particularly their mother [100,103,109] and will reject affection when the mother seeks to provide it but demand affection on their own terms [103]. For these children, keeping in control is a way of feeling safe because they have understandably come to believe that they cannot rely on others to look after them [100]. Similarly, children may be prematurely self-reliant and self-contained and not seek comfort or assistance from parents [82,109,119]. When distressed, such children may suppress the external manifestations of their disturbance [120] because they do not expect that anyone would respond to their cries or because they do not want to risk being rejected [109]. Thus, for example, some young children will quietly lie in bed after wakening and will wait (up to several hours) until someone comes to attend to them [78,121]. They may also exhibit self-soothing behaviours such as rocking, finger sucking, head banging or masturbation [103,118]. Self soothing behaviours are common in the general population; however, in children who have been abused or neglected, self soothing is more frequent [118] and in preference to comfort that their primary caregiver might seek to provide them with. Some children aggressively avoid intimacy with their mother and actively reject her [109,111] and they may consistently act in such a way as to attempt to make themselves undesirable to their mother [103]. Thus, children's past experiences of hurt in relationships result in them seeking to resist nurturing care from their mother [98]. There are other behavioural symptoms that children with a history of relational trauma may exhibit such as excessive clinginess [109], seeking to control their environment [103] or night disturbance [122] However, these symptoms are less problematic in terms of directly placing a barrier to the mother building a trusting relationship with her child and so will not be discussed.

It is important to recognize that the time of placement in an adoptive family is extremely stressful for children. Children form attachments to their caregivers even if they are abusive or neglectful and so loss of any caregiver is stressful [123]. Newly adopted children have not only lost previous caregivers and familiar environments but have also gained new caregivers and a new environment in which they have to learn to live. For children with a history of abuse or neglect, dealing with this stress can be particularly difficult, since not only are they likely to have an impaired regulatory system that disorganizes under stress [107] they also have not developed an attachment to the new caregiver to assist them in dealing with this stress [98].

It is vital for a child who has not developed a healthy attachment in infancy or whose attachment has been disrupted to become attached in order to heal and grow [124]. For a newly adopted child with a history of relational trauma, assisting them to trust and to build a secure attachment relationship with their mother can be a challenge. Nonetheless, adoptive parents are motivated to help their children with the hope that they can reverse damage and enable their children to achieve good outcomes in adult life [100]. This hope is well founded since there is evidence that early difficulties can be overcome [74,76,125] and that even the stress physiology of child can be altered by appropriate parenting [126]. In this context, breastfeeding is a parenting tool that may be used to assist child-maternal relationship development.

### Initiating adoptive breastfeeding

It is not to be expected that children who have experienced relational trauma will be willing or able to start breastfeeding immediately post-placement. The experience of mothers who have pursued breastfeeding with their adopted child is that building trust and attachment with their child, and slowly introducing the idea of breastfeeding, enables the establishment of a breastfeeding relationship.

An ongoing study of adoptive breastfeeding, being conducted by the author, has collected the experiences of adoptive mothers in initiating breastfeeding with their non-newborn children adopted via intercountry or domestic adoption. Many of the children in this study have histories of abuse or neglect. Thus far, the results indicate that, for most children, time spent building trust and attachment between themselves and their mothers is a necessary part of the process of working towards breastfeeding. It appears that a threshold level of trust and attachment is required before most children can contemplate breastfeeding. Under these circumstances, it can be helpful to picture the initiation of adoptive breastfeeding as "weaning to the breast" and a gradual progression in which the mother is able to help her child to be comfortable with intimacy with her and thus with breastfeeding.

Therefore, mothers who wish to breastfeed their adopted child are advised to instigate caregiving strategies that will build trust and attachment. This may include (but is not limited to) maximizing skin-to-skin contact, carrying the child frequently, providing massage, co-sleeping, co-bathing, hand feeding and responsive caregiving [127]. It is important for mothers to follow their child's lead in initiating close physical contact [98] and, while it is vital for them to be respectful of any distancing their child might employ in relation to physical contact, it is also necessary to be persistent in gently initiating close contact. It is the experience of adoptive families that children who push away their mother in the beginning of relationship development can often be persuaded with persistence that intimacy is desirable [125]. While initially children may, for example, not want to be held, may be stiff when held, may not give eye contact or may not want to co-sleep, gentle persistence on behalf of the mother may overcome each rejection and become another step closer to the comfort and trust required for breastfeeding to be possible. Thus, the reluctance of a child to immediately breastfeed (such as described by Riordan [5]) should not be taken to mean that breastfeeding is undesirable, inappropriate or impossible but understood as a normal and expected reaction to placement and as a starting point for relationship development.

In addition to attachment and trust building activities, it is often helpful for mothers to provide opportunities for their child to observe other children breastfeeding and to regularly offer their child the opportunity to suckle [128]. It appears relatively common for children to gradually move towards breastfeeding in a progression whereby they start by rejecting the breast altogether, then licking their mother's nipple when offered the breast, then suck very briefly at the breast and then move to full breastfeeding. The progression to breastfeeding will often be inconsistent and children will move backwards and forwards for some time before finally committing to full breastfeeding. Mothers have described how, during this time of transition, their child's forward or backward movement provides an indication of how well their child was doing in general with their child pulling back when feeling less safe/secure and moving forward when feeling more safe/secure.

In cases where children are still bottle feeding at placement, mothers frequently find it helpful to transition their child to breastfeeding by gradually making bottle feeding more like breastfeeding [128]. The sequence varies from situation to situation but the steps often used include: holding the child in a "breastfeeding position" while they are being bottle fed, using a slow-flow bottle teat, changing sides while bottle feeding and bottle feeding with the child skin-to-skin with the mother's bare breasts. In some instances, children are willing to suckle direct at the breast at this point. However, where children remain reluctant to breastfeed many mothers have had success with threading the tube of a breastfeeding supplementer (such as the Medela Supplemental Nursing System™ or the Lact-Aid^® ^Nursing Trainer) through a bottle teat or nipple shield which is then placed over the top of the mother's nipple so that the child obtains milk from the supplementer while sucking at the breast [128]. Mothers have also simply filled a bottle teat with milk and placed it over their nipple, which similarly allows the child to obtain milk while providing a barrier between mother and child that makes suckling at the breast tolerable for the child. When this technique is used, the teat or shield can be removed when the child is ready and direct suckling at the breast can proceed. It is also common for children to oscillate in their progression to breastfeeding with this technique.

Mothers need to be gentle, respectful and persistent in offering breastfeeding to their child and expect that it may take several months or even more than a year before their child is ready to breastfeed. Development of trust and attachment takes some time [74] and therefore it is not surprising that the progression to breastfeeding might require a significant time investment. It is extremely important that mothers not attempt to force their child to breastfeed, this simply does not work but can create an aversion to the breast and harm the development of trust between child and mother. It is also important to note that while most children with a history of relational trauma will be initially reluctant to breastfeed, once committed to breastfeeding children generally become avid breastfeeders who clearly gain a great deal from the intimacy and comfort of breastfeeding, as will be discussed.

### Children who seek breastfeeding

Although this paper has outlined why intimacy and breastfeeding are initially difficult for children with a history of abuse or neglect, paradoxically, there are also instances where newly adopted children seek breastfeeding from their mothers [129]. Adopted children seeking breastfeeding does not appear to be a rare occurrence and children from less than a year to more than 10 years of age at placement have indicated a desire to breastfeed [129]. Children have shown their mothers that they want to breastfeed by attempting to remove their mother's clothing to access the breast, taking advantage of being skin-to-skin with their mother to seek suckling or by verbally requesting breastfeeding [129]. In some cases, it is possible that children who have sought breastfeeding had been breastfed for a significant period of time by their birth mother and had a conscious memory of breastfeeding as being an appropriate activity between mother and child. In other instances this is not the case and it appears that children are remembering at a deep level: their infancy, the desire for attunement they were born with and an associated desire to suckle [129]. For these children, the movement to a nurturing environment with an adoptive family after having experienced abusive or neglectful caregiving may be a catalyst for this remembering. The stress involved in placement may also be involved in breastfeeding seeking behaviour via activating memories of early experiences [107] or via the impact of stress in causing regression in behaviour [129]. Understandably, their child wanting to breastfeed has surprised many mothers and while some have been willing to satisfy their child's desire, others have substituted bottle-feeding. Sometimes the desire of a child to suckle has been transitory but in other cases it has lasted for years [129]. The desire of children to breastfeed or have close contact with the breast can sometimes be misinterpreted as a mother projecting her desire to breastfeed onto her child [130]. However, children seeking breastfeeding has been widely reported in numerous contexts by mothers who had no intention of breastfeeding [122,129,131,132] and there is no evidence that this phenomenon is other than child-driven.

### The impact of breastfeeding

There are a number of ways in which breastfeeding affects adopted children and their mothers. Mothers who have breastfed their adopted children have described how, once breastfeeding is established, it provides a way in which they are able to comfort their children [128], and some have described observing both physical and emotional relaxation in their children during breastfeeding [128]. Abused or neglected children are often hyper-vigilant and have difficulty relaxing [124] but, as previously discussed, the hormonal release, skin-to-skin contact and consumption of milk involved in breastfeeding provides relaxation and comfort to children. In addition, for those children who experience chronic pain, breastfeeding offers pain relief [133]. Mothers whose child previously rejected comfort from them have stated that being able to provide comfort through breastfeeding was very important to them. Thus, breastfeeding can assist adoptive mothers to complete the attachment cycle with their child.

Breastfeeding may also assist children in dealing with stress. In Western societies it is common for children to use transition objects such as blankets or soft toys to help them cope in stressful circumstances. It is thought that use of transition objects represents the child's redirection of attachment behaviour to an inanimate object when the mother is not present [134]. However, breastfeeding is an example of attachment behaviour directed towards the mother within which the mother provides stress relief to her child through her own body. Adoptive mothers have described how their child has used breastfeeding and the related physical closeness to help them to cope in stressful situations [128] and to regulate themselves in a way reminiscent of maternal-newborn regulation [135]. Thus, when children have become disorganized or stressed they will seek breastfeeding as a way of connecting with their mother and centring themselves [128]. Mothers also commonly find that breastfeeding helps their children to go to sleep [127,128], something that is often difficult and stressful for newly adopted children [122,136].

The closeness and intimacy involved in breastfeeding may be important to children with a history of relational trauma for the very reasons that breastfeeding is often initially impossible for them. At placement both child and parent are alien to each other and it is only over time and through intimate interaction that the mother-child relationship develops [137]. Breastfeeding helps to forge mutually intimate relationships because characteristics of intimacy, such as reciprocity, harmony, trust, emotional closeness and skin-to-skin contact, are all part of the breastfeeding experience [138]. As previously discussed, children with a history of abuse or neglect often seek to be in control of relationships because they have had to be self reliant prior to adoption. However, there is a physical acceptance of the mother by the child as the child suckles at the breast, and it could be hypothesized that this physical acceptance flows out of an emotional acceptance of the mother. Intimate contact between mother and child also influences the physiology of both, resulting in the release of opioids (beta endorphins) in the brain of each, creating feelings of pleasure [27,69].

For those children who sought breastfeeding from their adoptive mother, allowing suckling, even if it only occurs on a few occasions, is something that mothers describe as being important to their child in demonstrating that they physically accept their child [129]. In addition, there are a number of children for whom seeking contact with the breast or breastfeeding has allowed them to express their vulnerability and to grieve over the loss of their birth mother [129,130]. For those children who seek breastfeeding the need appears to be a deep one and the experience of mothers is that meeting this need is beneficial [129].

Breastfeeding also provides an opportunity for mothers to provide some of the early care that their children may have been deprived of [129]. Brain development is experience dependent [139] and therefore it is possible that providing intimate maternal-child interaction might stimulate development of the relationship regulating right brain [107] in such a way to aid healing from early abuse or neglect [127]. There are many sensory and relational experiences involved in breastfeeding that children who have been abused or neglected may have been denied. For example, many abused or neglected children are deprived of nurturing touch and intimate social interaction; however, breastfeeding provides both of these [140]. Social interaction during breastfeeding can involve eye contact and adoptive mothers have found that breastfeeding is a time when extended eye contact between themselves and their child often occurs. Newborn babies show visual attentiveness during suckling and it is thought that this is related to the high probability for maternal social interactions at that time [13,141]. That newly adopted children often provide eye contact during breastfeeding may be related to these earlier mechanisms and may be significant in attachment development [142].

There are a number of changes in behaviour that mothers frequently report in their children after breastfeeding is initiated. These changes include: an increase in eye contact, calming and comfort, removal of body tension, emotional vulnerability, melding of the child to the mother's body, cuddliness, an increase in the child's desire to be with their mother and in older children, gentle behaviours reminiscent of the actions of young babies (e.g. placement of their fingers in their mother's mouth, stroking of the mothers face) [128]. Some adoption professionals have found that an "in arms" environment where the child is cradled like an infant face-to-face, eye-to-eye can provide environmental triggers that allow children with a history of relational trauma to access previously dormant attachment needs, feelings and behaviours [102]. Breastfeeding has not been previously considered as potentially part of this "in arms" experience; however, suckling at the breast would heighten the sensory experience of child-mother closeness as well as provide stress relief and comfort. Therefore, the changes that parents observe in their children after breastfeeding is initiated may be related to environmental triggers associated with the "in arms" experience noted by adoption professionals.

Breastfeeding may also assist adoptive mothers to care for their child. As previously described, breastfeeding physically connects mother and child and promotes maternal sensitivity. The time of placement of an adopted child is often difficult and stressful for parents [143]. Adoptive parents often strive to feel entitled to care for their child in the face of societal attitudes that regard adoptive families as less authentic than biological families [144,145]. In addition, as described, newly adopted children may present with some challenging behaviours that make caring for them difficult [146]. Breastfeeding, however, may provide some stress relief to adoptive mothers, help them to feel entitled to parent, build their confidence in mothering and assist in healing any grief that may be present as a result of infertility [147,148]. It is important to note that adoptive mothers come to parenting at a disadvantage not only because their child's life experience may make it difficult for him/her to accept nurture but also because they do not experience the hormonal priming for mothering that occurs during and immediately after pregnancy [149]. However, breastfeeding can assist adoptive mothers by stimulating the release of oxytocin, prolactin and cholecystokinin and requiring mothers to maintain physical proximity to their child. Adoptive mothers have reported that breastfeeding resulted in a softening of their own attitude towards their child and a feeling of oneness with their child [128]. The development of attachment is a cooperative effort between mother and child, with children becoming attached to emotionally available, sensitive caregivers [109,150]. Since breastfeeding promotes maternal sensitivity it will also promote attachment development.

The process of working towards breastfeeding may also assist mothers to become attuned to their child [128]. It is sometimes the case that a newly placed child can appear to be doing quite well, but when their mother seeks intimacy with them, a difficulty with closeness is revealed. However, in the process of working towards breastfeeding, mothers have described how they became highly sensitised to their child's emotional well being [128]. Thus, even mothers whose children never breastfed have stated that they appreciate the sensitivity that working towards breastfeeding developed in them and that they feel that their attempts to facilitate breastfeeding assisted the development of the child-mother relationship [128]. It should be noted that if breastfeeding is not possible, providing food directly to the child (hand feeding) or bottle-feeding may assist in replicating somewhat the early-expected experience of nurture through food [127].

### Case histories

The following case histories present the experiences of mothers who have breastfed their adopted children. The mothers in these case histories gave permission for their stories to be shared and pseudonyms are used for privacy reasons.

#### LiJun

LiJun was adopted from China at the age of three years. She had been abandoned as a young baby and placed in an orphanage where she lived until adoption. Staffing levels in the orphanage were low and while it appeared that the carers had a genuine affection for LiJun they could not provide individual care or a stimulating environment. Thus, at placement LiJun was developmentally delayed and exhibited other symptoms associated with long-term institutionalisation. The transition to her adoptive family was abrupt and LiJun was overwhelmed and very distressed by what was happening to her. However, the morning after placement she started interacting with her new family showing an immediate preference for her adoptive father. While LiJun appeared generally happy during the day she had great difficulty going to sleep and would also frequently wake during the night at which time she expressed anger or sadness in her cries. During her night distress she vehemently rejected her mother but would accept comfort from her father.

Upon return home, her mother began to attempt to transfer LiJun's preference to herself. She persisted with initiating physical closeness with LiJun and carried her in a sling, co-slept, co-bathed and hand fed her and slowly LiJun warmed to her mother. However, LiJun's night distress remained. It would take up to three hours for LiJun to go to sleep and during the night she would wake 6–12 times. Often her mother needed to take LiJun from the bed and walk and carry her in order for her to go back to sleep.

Several weeks post-placement, LiJun's mother relactated and began offering breastfeeding. Her mother's motivation to relactate initially arose out of a desire to provide breastmilk in order to prevent illness; she was concerned that LiJun's emotional state left her vulnerable to infection and that illness would be another burden on a child who was struggling to cope. LiJun drank the milk her mother produced in a cup but attempts to introduce a bottle failed because LiJun did not appear to know how to suck on the teat. Nevertheless, her mother showed LiJun pictures of breastfeeding couples, offered the breast each day and after a couple of weeks LiJun licked her mother's nipple and then sucked very briefly for the first time. Thereafter, LiJun would suck for less than a second on her mother's nipple every few nights. Two months post-placement, LiJun's mother was carrying her in a sling as she attempted to soothe her to sleep when LiJun started sucking on her mother's neck. In response to this her mother laid her down in the sling, offered the breast and LiJun attached to the breast, suckled and fell asleep. For two months after this LiJun suckled to sleep in the sling at night on a semi-regular basis but she would not breastfeed at any other time and would not breastfeed if she had had a challenging day or was otherwise unsettled. Although LiJun was breastfeeding, it was sometimes obviously difficult for her to do. However, five months post-placement and two months after beginning to breastfeed to sleep, LiJun abruptly started to seek breastfeeding for comfort and reassurance day and night. LiJun then breastfeed up to 12 times a day and could be instantly comforted by breastfeeding on her still frequent night waking (which continued for two years post-placement). Her mother feels that LiJun used breastfeeding as a way of obtaining comfort and reassurance, as a touch point to herself and to aid self regulation when circumstances were overwhelming. Now three years post-placement LiJun continues to breastfeed to sleep at night but rarely seeks breastfeeding at other times. Her mother feels that the acceptance of breastfeeding paralleled LiJun's acceptance of herself as her mother and that it has aided her daughter in healing.

#### Jacqueline

Jacqueline was adopted from China at five years of age. She had been abandoned at the age of 18 months at which time she had a life threatening congenital heart defect. After abandonment, she lived in an orphanage for 2.5 years before heart surgery was performed, involving six weeks hospitalisation without any contact with caregivers. Post surgery Jacqueline was placed in foster care for eight weeks and during this time began to talk for the first time since her abandonment. Jacqueline experienced multiple transitions and multiple separations from multiple caregivers during her first five years but after a final nine months in the orphanage she was placed for adoption. Her mother states that post-placement Jacqueline presented as a generally happy child who was energetic and eager to please; however, she was also indiscriminately affectionate and developmentally delayed, hoarded food, wanted to be self-sufficient and was afraid of and had difficulty recognizing emotions.

After placement in her adoptive family her mother sought to parent Jacqueline in such a way as to promote attachment and so kept her in close physical contact, co-bathed and co-slept with her and used massage to provide nurturing touch. Jacqueline would periodically express some of the anger she felt as a result of her past hurts in what her mother describes as a "rage." After one such "rage" she indicated a need to suck. She sucked hard on her mother's fingers, nose and face before finding her breast, latching on, suckling and falling asleep. Her mother was very surprised by Jacqueline's desire to breastfeed but perceived this need to suck as being a "primal urge" coming from deep within her, not something that was occurring at the conscious level, and felt that it would be helpful to allow her to suckle. Her mother believes that Jacqueline must have been breastfed by her birth mother because of the way she latched and suckled as an experienced breastfeeder and thinks that she was seeking to re-enact earlier times of comfort and nurture. After this incident, Jacqueline continued to initiate breastfeeding sporadically; up to several times a day and as little as once every a fortnight. Jacqueline was content to suckle without milk being present but her mother relactated after breastfeeding had continued for a few months with the aid of a breast pump. Breastfeeding was used by Jacqueline as way of finding of comfort and security and was sometimes associated with Jacqueline "playing baby." The frequency of breastfeeding generally decreased over time until weaning at 2.5 years post-placement. Jacqueline showed a dramatic acceleration in emotional, physical and cognitive development during the first two years post-placement. Jacqueline's mother views her acceptance of her child's desire to breastfeed as helpful and feels that it has promoted healing, attachment and belonging in her adoptive family and has helped her child to feel secure.

#### Catherine

Catherine was born full-term with a life threatening condition that had been detected antenatally. As a self-protection mechanism, her parents emotionally withdrew from her during pregnancy and although surgery post-birth was successful and Catherine was no longer at risk, her parents remained detached from her. Thus, Catherine spent the first four months of her life in hospital with minimal interaction with her parents or other adults. Catherine did not gain weight in the early weeks of life, was diagnosed as failure to thrive and had a gastrostomy tube inserted. The insertion of the gastronomy tube did not result in weight gain but reduced the amount of human contact Catherine experienced because feeding did not require her to be held. Catherine was not provided with emotionally sensitive care or affection during her time in hospital. At four months of age and weighing 3.2 kg her parents relinquished her for adoption and she was placed in foster care. At the time she entered foster care, Catherine had developed an aversion to interaction with others and would turn away if spoken to and stiffen if touched. However, her foster mother provided her with responsive care and Catherine formed an attachment to her. Catherine was in foster care for ten weeks before being placed for adoption at 6.5 months of age. Post-placement for adoption, Catherine grieved her foster mother deeply and although she tolerated her adoptive father she rejected her adoptive mother and would turn her head and push her mother away if she attempted to carry her facing towards her. Her adoptive mother had previously breastfed other adopted children placed as newborns but her early offers of breastfeeding to Catherine were strongly rejected. Catherine weighed only four kilograms at placement and had a strong aversion to bottle-feeding. She would take only small amounts of milk via a bottle and only if she was held upright and facing away from the caregiver. Sucking was not pleasurable for Catherine, she did not suck on a pacifier or her thumb and would gag and vomit if her mother attempted to reinsert the bottle teat after Catherine had pushed it out of her mouth. Catherine's mother worked towards promoting the emotional and physical health of her child by committing to carrying Catherine in a sling whenever possible, being responsive to her and always remaining close to her. Within a few weeks Catherine developed a preference for her adoptive mother and would not tolerate any physical separation from her. Her mother also worked to overcome Catherine's oral aversion and changed her infant formula to one more pleasant tasting to that previously used and persisted with bottle feeding. As a result, Catherine developed a love of sucking. At this time Catherine also began to gain weight (though her energy intake had not increased), which allowed for the removal of the gastronomy tube. When Catherine's health had improved her mother decided to reattempt breastfeeding. Catherine was being bottle-fed very frequently at this stage and her mother decided that she would always be the one to feed her and that she would attempt the transition to breastfeeding by making bottle-feeding progressively more like breastfeeding. Thus, her mother used a short bottle, which made it easier to turn Catherine towards her as she fed and would switch sides during feedings. She also allowed Catherine to suck at the bottle after all the milk had been consumed for as long as she wanted to, which was often for many minutes. Once she was bottle-feeding well her mother threaded the tube of a breastfeeding supplementer through a bottle teat and held this over her nipple while Catherine sucked and obtained milk from the supplementer. When she felt that Catherine was ready, she replaced the bottle teat with a nipple shield with the supplementer tube placed through the end and finally she removed the nipple shield so that by her first birthday Catherine was suckling direct at the breast. Once breastfeeding began, her mother noticed an increase in Catherine's confidence and security. Thus, when Catherine was hospitalised shortly after starting breastfeeding, she not only surprised her doctors by her speed of recovery but by the marked improvement in the way she coped with the stress of medical procedures. Previous post-adoption hospitalisations had seen Catherine show terror whenever she was approached by someone wearing a white coat but on her first hospitalisation after breastfeeding initiation she spent much of the time while in hospital breastfeeding and allowed herself to be examined without showing distress. Post-breastfeeding initiation Catherine also experienced acceleration in developmental progress and a detachment from bottles to which she had developed an emotional connection. By the time of weaning at two years of age, Catherine had caught up physically and developmentally and was using her mother as a secure base when exhibiting normal explorative behaviour.

#### John

John was born cocaine and alcohol addicted and expressed behaviours associated with prenatal drug abuse including: irritability, hypersensitivity to stimulation and persistent, protracted crying. On hospital discharge at six days of age, he was placed in foster care with the intention that he would eventually be placed in the care of his father. During the first few weeks of his life his foster mother reported that he was "screaming" or sleeping most of the time. Swaddling, close physical contact and sometimes skin-to-skin contact were used by John's foster mother to calm him. Many aspects of normal life were stressful to John including stimulation from noise, light or touch. Eye contact or a kiss from his foster mother would result in a bout of intense crying. When being bottle fed, John would become distressed and arch his back; however, on several occasions when his foster mother held him swaddled on her skin to sleep she awoke to find him calmly latched and suckling at the breast. His foster mother did not pursue breastfeeding at this time because she wished to support the placement of John with his father.

Over the next few months, John's foster mother continued to care for him in such a way as to reduce his distress as much as possible and he gradually calmed somewhat and became less unhappy. Nonetheless, he remained a tense and physically stiff baby who would not relax in his foster mother's arms and did not like facing her. When John was six months old permanency planning switched from placement with his father to adoption by his foster family and his mother decided to attempt to breastfeed him. Using a breastfeeding supplementer she offered John the breast and he accepted it immediately. His mother fed him at the breast consistently and noticed changes in his behaviour. John became much more physically relaxed and stopped holding his fists tightly clenched, he began sleeping much better and started to become more interested in what was going on around him. He also began to turn towards his mother when she held him and would physically meld into her, even falling asleep as she carried him, something he had never previously done. He commonly fell asleep breastfeeding. Within two weeks of breastfeeding initiation John also began to enjoy eating solid food, to sit alone and roll. However, most significant for his mother was that, in her words, he changed from "a rigid, screechy baby who didn't like to be held, to a calm, happy, snuggly infant" and the changes were so rapid, over a period of only a few weeks, that his mother is convinced that breastfeeding was involved.

### Limitations of breastfeeding

Although there is evidence that breastfeeding can assist children with a history of relational trauma to heal, breastfeeding is not a panacea and will not solve every difficulty. Thus, while breastfeeding may comfort children and assist in the development of the attachment relationship, this does not mean that every adopted child who breastfeeds will develop a secure attachment with their mother. Building a trusting relationship with a child who has been abused or neglected can be extremely difficult and developing a secure attachment may be impossible for some children. In some cases, day-to-day life can be so difficult for children with histories of relational trauma and their families that assistance from mental health professional will be required. In addition, even where children with a history of relational trauma are able to form secure attachments with their caregivers, early abuse or neglect may have a permanent impact on brain functioning [107,151]. However, children who have formed secure attachments with their adoptive parents will be best able to function in spite of deficits. Thus, breastfeeding is one tool that mothers can use to provide nurturing as they seek to develop trust and attachment with their child and should be used with other parenting strategies that will promote attachment. Nonetheless, though families still face many challenges as a result of their child's history, mothers who have initiated breastfeeding with their child have universally expressed that breastfeeding was helpful to them and their child.

Although the potential impact of breastfeeding is large, it is not possible to make a blanket recommendation that breastfeeding be attempted in all cases of adoption because of the individual circumstances of mothers and children. Some adoptive mothers will not be comfortable with the idea of breastfeeding their child and even for those who express a desire to breastfeed preplacement, the reality of caring for a newly adopted child may divert attention from efforts to breastfeed post-placement. In addition, because a certain level of attachment and trust is usually required for children to consider breastfeeding, children who have been very badly hurt may find breastfeeding impossible.

Breastfeeding focuses on building the relationship between child and mother and some could consider that this would be detrimental to the child-father relationship. Certainly, working towards breastfeeding and breastfeeding itself may result in children initially developing a deeper relationship with their mother than their father. However, it should be remembered that in cases of adoption, the child-parent relationship is being built from the beginning and that in intact birth family situations relationship development also begins with the mother [124]. In addition, in children with a history of relationship trauma, once they have built a relationship of trust with one person it may be easier for them to form trusting relationships with others [152,153]. Thus, while the process of working towards breastfeeding and breastfeeding itself may initially focus relationship development on the mother, it may also lead to development of a better quality of relationship with the father.

### Issues for practitioners to consider

#### Supporting mothers

Mothers who wish to breastfeed their adopted child are in need of support, encouragement and education. As described, it is common for children to initially reject breastfeeding and their mothers, and, particularly for women who have a history of infertility, being rejected by their long-awaited child can be devastating. Practitioners have a vital role in supporting and educating mothers so that they are able to understand and withstand rejection from their child. Such education may include informing mothers about the importance of not taking rejection personally or accepting rejection at face value and emphasising the need to avoid responding to rejection by withdrawing nurturing [98,99,115,119]. Education on the root of rejecting behaviour may also assist mothers to feel empathy towards their children [98], which will allow mothers to provide the sensitive care that enables attachment development [115,154] and to persist with working towards breastfeeding. Mothers may be encouraged to be gently persistent in providing nurturing without expecting reciprocity but with the knowledge that research has found that most children will eventually respond positively to nurturing [98]. Practitioners may also be able to assist mothers in locating networks that will support them in providing sensitive parenting and breastfeeding, since in a Western context parenting practices that promote attachment development are contrary to the cultural norm [155].

#### At what age can children breastfeed?

The issue of breastfeeding non-infant children is contentious [156]. However, breastfeeding has been initiated in children up to at least four years of age at placement and, as discussed, children who have sought breastfeeding have been up to more than ten years of age. In addition, children with a history of relational trauma are likely to be much younger emotionally than their chronological age would indicate [100], and providing nurturing care and intimacy more commonly considered suitable for younger children can be beneficial [100,127]. It should also be considered that Western societies have a particularly short term view of breastfeeding while anthropological research suggests that breastfeeding is a normal activity for children aged up to seven years of age or more [157]. Nonetheless, prevailing societal attitudes make it advisable for practitioners to suggest to parents that they need to be careful to whom they reveal that their child is breastfeeding if the child is more than two or three years of age.

#### Talking about adoptive breastfeeding

The possibility and potential impact of breastfeeding is something about which few are aware; practitioners have a role in promoting and educating others about adoptive breastfeeding. It should be noted that adoptive parents are often highly motivated and committed to helping their child [72] and it should not be assumed that families will be "put off" from breastfeeding because it may be challenging. Thus, it is appropriate to discuss breastfeeding with prospective adoptive parents.

#### Breastfeeding of foster children

Although adoptive breastfeeding has been the focus of this paper it is worth noting that foster children are sometimes breastfed by their foster mothers. Foster breastfeeding sometimes proceeds with the permission and support of the relevant authorities [133], sometimes without [158], and the possibility of gaining permission to breastfeed a foster child varies widely between locations. However, those considering breastfeeding a foster child without approval should be aware that children have been removed from foster families because they were being breastfed by their foster mother. Nonetheless, it can be argued that breastfeeding should be considered in foster care situations where it is anticipated that the child will be in care for six months or more [133].

### Breastmilk

This paper has considered the emotional impact of breastfeeding a child with a history of abuse or neglect; however, the unique properties of breastmilk also confer positive immune and growth factors to children [8]. In addition, newly adopted children may have increased vulnerability to infection as compared to non-adopted children for several reasons. There is evidence that early trauma can have long term negative effects on immune function [159] and that attachment insecurity leaves individuals more susceptible to disease [160]. In addition, children who are under emotional stress (such as all newly placed adopted children) may be more vulnerable to infection [161,162]. Children adopted via intercountry adoption have experienced a drastic change in environment that may include exposure to pathogens to which they were previously unexposed [163], and likewise a high proportion of children adopted from foster care have health issues [93]. Thus, the health enhancing properties of breastmilk may be particularly important to adopted children.

## Conclusion

Breastfeeding has the potential to promote the development of the child-maternal attachment relationship in vulnerable adoptive dyads. The impact of breastfeeding arises out of aspects of the physical act of breastfeeding that have been largely overlooked in comparison to the nutritional and immune properties of breastmilk. However, the impact of breastfeeding as observed in cases of adoption has relevance to all breastfeeding situations and this deserves further investigation. In particular, there may be applicability of the experience of adoptive breastfeeding to other at risk dyads, such as intact families with a history of intergenerational relationship trauma.
